# A Case of Rapidly Progressive Coccidioidal Meningitis in an Immunocompetent Patient

**DOI:** 10.7759/cureus.28643

**Published:** 2022-08-31

**Authors:** Mariam Fatima, Reema S Patel, Jamie R Brunworth, Krisha A Gupta, Wesley E Roach, Paige L Webeler, Cherie R Mundelein, Mohamed Mansour

**Affiliations:** 1 Osteopathic Medicine, Nova Southeastern University Dr. Kiran C. Patel College of Osteopathic Medicine, Clearwater, USA; 2 Osteopathic Medicine, Nova Southeastern University Dr. Kiran C. Patel College of Osteopathic Medicine, Ocala, USA; 3 Internal Medicine, Nova Southeastern University Dr. Kiran C. Patel College of Osteopathic Medicine, Fort Lauderdale, USA; 4 Osteopathic Medicine, Nova Southeastern University Dr. Kiran C. Patel College of Osteopathic Medicine, Fort Lauderdale, USA; 5 Internal Medicine, Nova Southeastern University Dr. Kiran C. Patel College of Osteopathic Medicine, Clearwater, USA; 6 Internal Medicine, Nova Southeastern University Dr. Kiran C. Patel College of Osteopathic Medicine, Ocala, USA; 7 Emergency Medicine, Nova Southeastern University Dr. Kiran C. Patel College of Osteopathic Medicine, Fort Lauderdale, USA; 8 Critical Care Medicine, AdventHealth Ocala, Ocala, USA

**Keywords:** infectious disease diagnosis, coccidioidal meningitis, coccidioidomycosis, fungal meningitis, meningitis

## Abstract

A 50-year-old male with a history of a dull headache and neck pain for a few weeks presented to the ER with complaints of progressive weakness and difficulty walking. Physical examination revealed a lethargic, confused patient with abnormal tremors at rest. Initial lab work was significant for elevated hemoglobin, hematocrit, and hyponatremia. Additionally, CT imaging was significant for prominent ventricles. Several serologies and polymerase chain reaction (PCR) tests were ordered to determine the etiology of the patient’s meningitis. On day 10 of admission, serology results returned positive for *Coccidioides* antibodies. The patient was started on an IV fluconazole treatment and underwent a ventriculoperitoneal shunt and Ommaya reservoir placement procedure. Cases of coccidioidal meningitis are rarely noted in recent literature. We present this clinical case of coccidioidomycosis dissemination into the central nervous system (CNS) to highlight the rare localization of the fungal infection in a baseline immunocompetent patient.

## Introduction

Coccidioidomycosis is a fungal infection caused by the soil-dwelling fungus *Coccidioides*, a species native to the southwestern United States and Central America. Inhalation of the fungal arthroconidia causes particle deposition into the lungs, where the arthroconidia transform into spherules and eventually burst, releasing endospores into the tissues. This burst of endospores causes a host response of acute inflammation, and in cases of immunocompromise, the spherules may travel to extrapulmonary locations. While 60% of infections will remain asymptomatic, some common symptoms include fatigue, cough, fever, shortness of breath, headache, night sweats, joint pains, and rash. Adults over age 60, pregnant patients, immunocompromised hosts, and those of African and Filipino backgrounds are known to be at the highest risk for manifesting symptoms of the disease [1]. Although it most commonly localizes in the lungs, coccidioidomycosis can also spread to the central nervous system (CNS), skin, bones, and joints. Among these sites, dissemination to the CNS is quite uncommon but has the highest mortality [2].

Diagnosis of coccidioidomycosis is often made via serology and radiographic imaging in endemic regions. The detection of antibodies in the blood is the most common method of diagnosis, but other fluids such as cerebrospinal, pleural, or joint aspiration can also be used [2]. For patients with the non-disseminated disease, *Coccidioides* infection is usually self-limiting over months without treatment. However, in more severe cases, antifungal medication can be required [2].

*Coccidioides* infection is geographically influenced, and most cases in the United States are reported in Arizona, Nevada, and California. Cases are uncommonly reported outside of these Southwest states. Additionally, only about 1% of patients with coccidioidomycosis developed more severe complications with pulmonary and CNS manifestations [3]. With an exceedingly rare confluence of factors, our patient contracted coccidioidal meningitis outside of the commonly expected states and experienced rapid onset neurologic dysfunction despite previous immunocompetent status. This case demonstrates that although rare, coccidioidal meningitis should remain in the differential of a patient with altered mental status and evidence of meningitis, even if the patient is immunocompetent.

A legally authoritative representative provided written informed consent for patient information and images to be published.

## Case presentation

Presentation

We present a case of a 50-year-old Caucasian male who arrived at an ED in Central Florida with complaints of a persistent dull headache for the past few weeks and bizarre behavior reported by his wife, including progressive weakness, difficulty walking, and struggling with word recall. The patient also had associated back and neck pain that had been ongoing since a fall from his camper two months prior. The patient was walking down steps when he slipped on a wet surface and experienced a four-foot fall, landing on his back. However, there was no loss of consciousness or head trauma from this incident. After the fall, the initial workup revealed only cervical degenerative disc disease. The patient was discharged with muscle relaxants. After this fall, the patient pursued chiropractic therapy for cervical neck adjustments and was recommended several nutritional supplements for recovery. Social history was significant for travel across the southern United States due to his occupation as a welder. Prior to this hospitalization, the patient’s hobby was bodybuilding. He admitted to several years of illicit anabolic steroid use for performance enhancement during workouts.

Assessment

In the ED, the patient’s initial vitals showed a heart rate of 89 beats per minute, a blood pressure of 142/104 mmHg, oxygen saturation of 99%, a respiratory rate of 18 breaths per minute, and a temperature of 97.6°F. In addition, the initial labs were significant for an elevated WBC count of 13.8 x 10³ per microliter, elevated hemoglobin of 20.2 g/dL, elevated hematocrit of 56%, and platelet count of 212 x 10³ per microliter. The only significant electrolyte abnormality was a sodium level of 123 mmol/L. Upon physical exam, the patient was lethargic and in no acute distress, with a grade 3/6 systolic murmur in the mitral focus. An eye exam revealed an abnormal arteriole/venule ratio in the retina, negative papilledema, and pupils were equally round and reactive to light. Lungs were clear to auscultation bilaterally. He had a normal range of motion and strength in the upper and lower extremities, and his skin and lymphatics exam revealed no irregularities. On the neurologic exam, the patient was confused, mumbled answers to the questions, and had abnormal tremors at rest. He was minimally interactive, responded very slowly to questions, and had altered mental status. An ultrasound of the carotid artery was negative for hemodynamically significant arterial stenosis. A CT head without contrast showed no acute intracranial hemorrhage but did show prominent ventricles with likely transependymal fluid migration posteriorly. The urine drug screen was negative.

Management

The patient was admitted to the hospital due to his altered mental status of unknown origin, with suspicion of meningitis. Initial laboratory workup included serology testing for SARS-CoV-2, neuronal nuclear IgG, influenza A PCR, influenza B PCR, myelin basic protein, respiratory syncytial virus (RSV) PCR, tetanus antibodies, Toxoplasma gondii PCR, and Varicella zoster PCR. An HIV panel, cerebrospinal fluid (CSF) analysis for amoeba, Cryptococcal antigens, herpes simplex virus (HSV) 1 and 2 IgM antibodies, HSV PCR, West Nile virus IgM and IgG antibodies, and Venereal Disease Research Laboratory (VDRL) screen for syphilis were also completed. At this point in the hospital course, phlebotomy with an erythropoietin check was ordered for concern of polycythemia vera due to the patient’s elevated hemoglobin levels, and neurology was consulted for suspected diffuse encephalopathy. There was also a suspicion of paraneoplastic syndrome. A bone marrow biopsy and therapeutic phlebotomy were performed due to the significantly increased hematocrit and hemoglobin levels. Nephrology was consulted for severe hyponatremia, for which normal saline at 100 milliliters per hour (mL/hr) and fluid restrictions were put in place. Neurosurgery did not recommend any surgical intervention at this time. Ampicillin, ceftriaxone, and vancomycin were started for empiric antibiotic coverage, dexamethasone for anti-inflammatory coverage, and acyclovir for possible HSV meningitis.

The patient’s CSF analysis revealed clear fluid with a critically elevated total protein of 307 mg/dL, critically low glucose of 32 mg/dL, and elevated WBCs at 42 cells/microL. Gram stain and culture of the CSF fluid were also negative. These results effectively ruled out bacterial meningitis as a cause of this patient’s symptoms, narrowing the differential to viral or fungal etiologies. HSV PCR testing of the CSF was negative; thus, acyclovir was discontinued. A QuantiFERON-TB Gold (QFT) and Coccidioides serology were ordered. 

**Figure 1:**
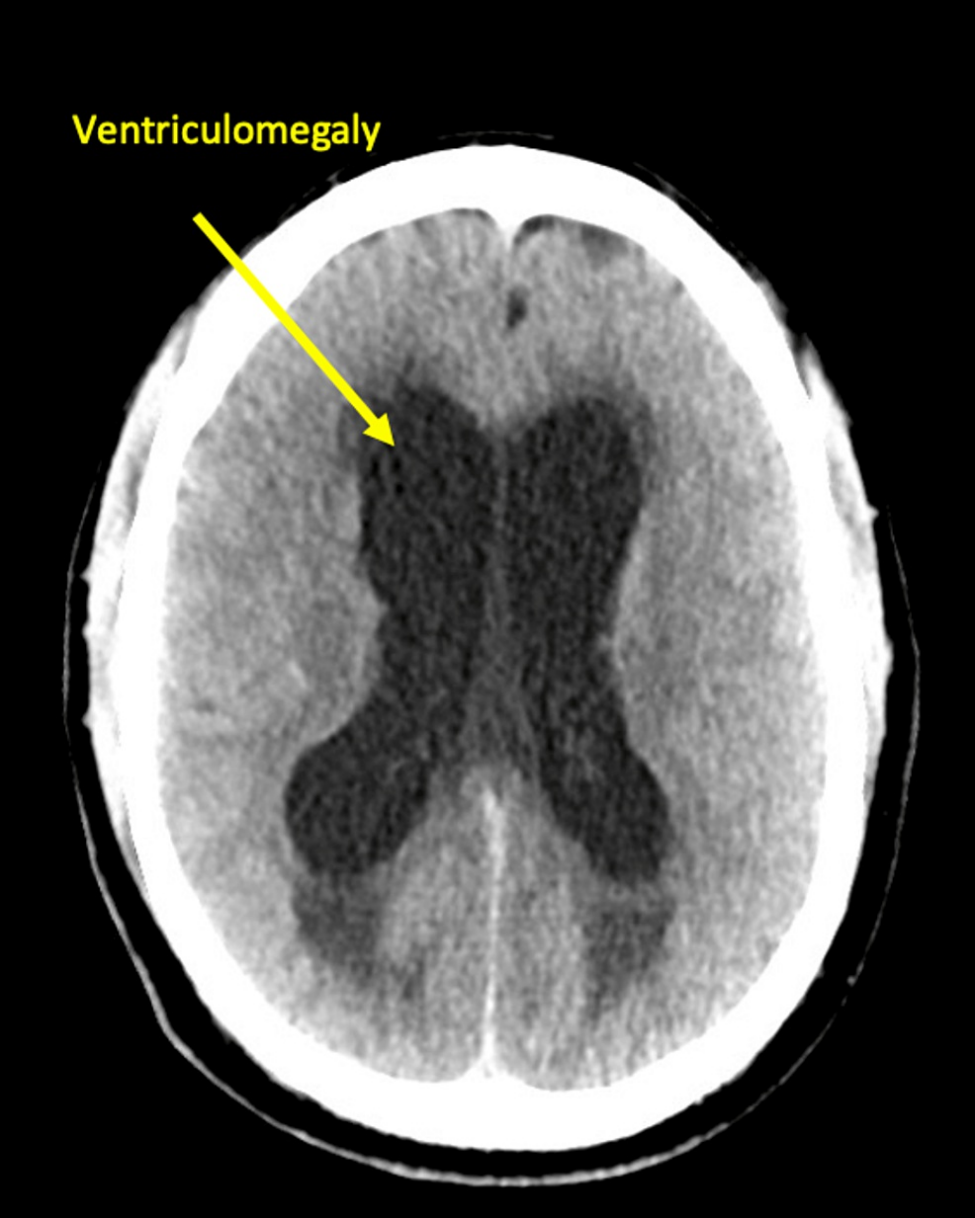
CT head without contrast on day 10 of admission revealed worsening ventriculomegaly with some transtentorial mass effect and signs of suspected cerebral edema.

At this point in the hospital course, serum sodium had increased to the normal range, and the patient’s mentation was significantly improved from his baseline at admission. He had undergone therapeutic phlebotomy, which lowered his hemoglobin and hematocrit to normal levels. His erythropoietin was within normal limits, and a bone marrow biopsy ruled out common neoplastic causes of polycythemia. The patient’s condition continued to improve gradually; however, he complained of muscle stiffness in his shoulders and neck. Empiric antibiotics were continued, and his sodium levels remained within normal limits. Polycythemia at this point was theorized to be due to his use of anabolic steroids as opposed to a neoplastic origin. Ampicillin was discontinued at this point, and the patient remained on a regimen of vancomycin and ceftriaxone. Due to an acute increase in his confusion, a repeat MRI of the brain and neck was ordered, which indicated no significant changes from the imaging four days prior. An HIV test was ordered, the results of which were negative. A magnetic resonance (MR) venogram was ordered for suspicion of possible cerebral venous thrombosis due to his initial hyperviscosity and polycythemia; however, this study returned negative as well. A CT scan of the abdomen and chest with and without contrast was ordered to evaluate for possible infectious sources. However, both came back negative. Laboratory testing for arsenic and lead levels were also negative. Neurology performed an electroencephalogram (EEG) which showed generalized slowing, nonspecific for any particular pathology but rather pointed to diffuse cerebral dysfunction. On day nine of admission, serum sodium level decreased abruptly to 120 mEq/L despite continued IV normal saline. A 15 mg dose of the aquaretic tolvaptan was administered, and serum sodium levels were rechecked in tight intervals to ensure adequate response. Sodium levels steadily increased, and another 15 mg dose of tolvaptan was given the next day. The patient’s mentation seemed to improve with the restoration of serum sodium levels, and he was noted to be much more alert with the resolution of headache and neck pain. On day 10 of admission, the QFT was confirmed negative, while anticoccidial complement fixation (CF) antibody titers were 1:16, confirming the diagnosis of Coccidioidal meningitis. Therefore, vancomycin and ceftriaxone were discontinued, and fluconazole was started at a dose of 800 mg every 24 hours. Due to evidence of worsening hydrocephalus, a repeat CT scan of the brain was ordered, revealing interval worsening ventriculomegaly with some transtentorial mass effect and signs of suspected cerebral edema when compared to the reference exam, but no acute intracranial hemorrhage was noted (Figure 1). 

**Figure 2:**
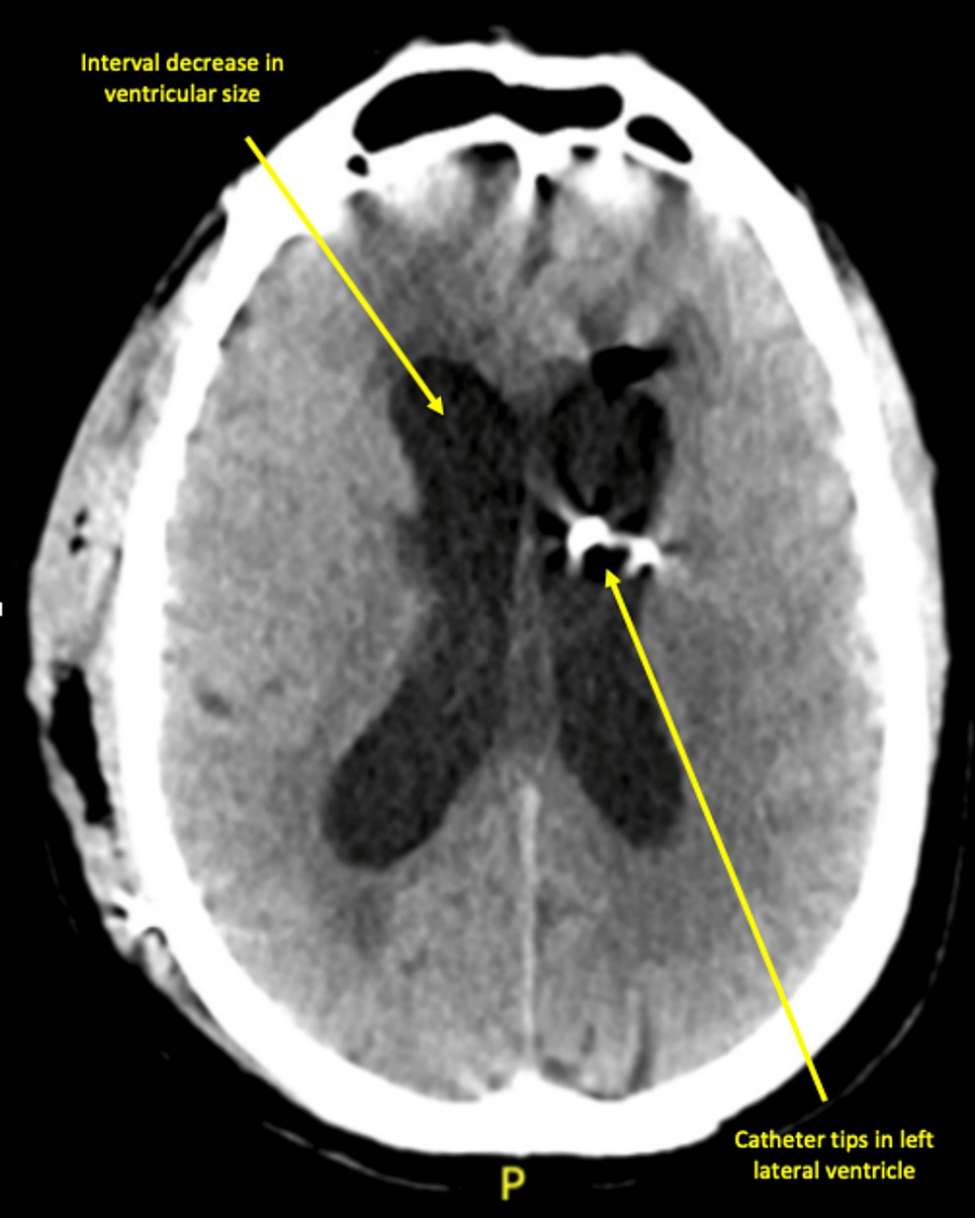
CT head without contrast on day 19 of admission revealed an interval decrease in ventricular size and catheter tips in the left lateral ventricle. “P” shown in the figure represents the posterior aspect of the head.

Neurosurgery was re-consulted for possible surgical intervention at this time and planned for insertion of an Ommaya reservoir. This ventricular access implant allows for access to CSF and eases the administration of medications. A non-programmable medium pressure ventriculoperitoneal (VP) shunt was also inserted into the left lateral ventricle to diverge CSF [4]. The neurosurgery team cautioned that in the case of coccidioidomycosis meningitis, there might be a high likelihood of future shunt obstruction due to increased protein and debris from the infection. Therefore, the plan for this two-step procedure was to be postponed until the CSF was cleared of fungal infection in an attempt to avoid future complications. By day 12 of admission, the polycythemia, hyponatremia, and metabolic encephalopathy had vastly improved. Considering the patient’s persistent prominent ventricles, a plan was made for a lumbar puncture one-week post initiation of antifungal therapy. Fluid analysis from the procedure revealed clear fluid with an elevated total protein of 189 mg/dL, low glucose of 55 mg/dL, and elevated WBCs at 283 cells/microL, which were predominantly lymphocytes. On day 19, a CT head without contrast revealed worsening hydrocephalus and a component of transependymal fluid migration. The patient was prepped for surgery, and the neurosurgery team inserted a VP shunt and Ommaya reservoir. The patient tolerated the procedure well and had no unanticipated events or complications. Postoperatively, the patient was doing well overall, with noted improvements in strength and speech, except for a minor headache. Imaging revealed improvement in ventriculomegaly (Figure 2). After the 14-day course of IV fluconazole was completed, the patient was discharged from the hospital on an indefinite course of oral antifungal medication, along with instructions to follow up with outpatient neurosurgery.

**Figure 3:**
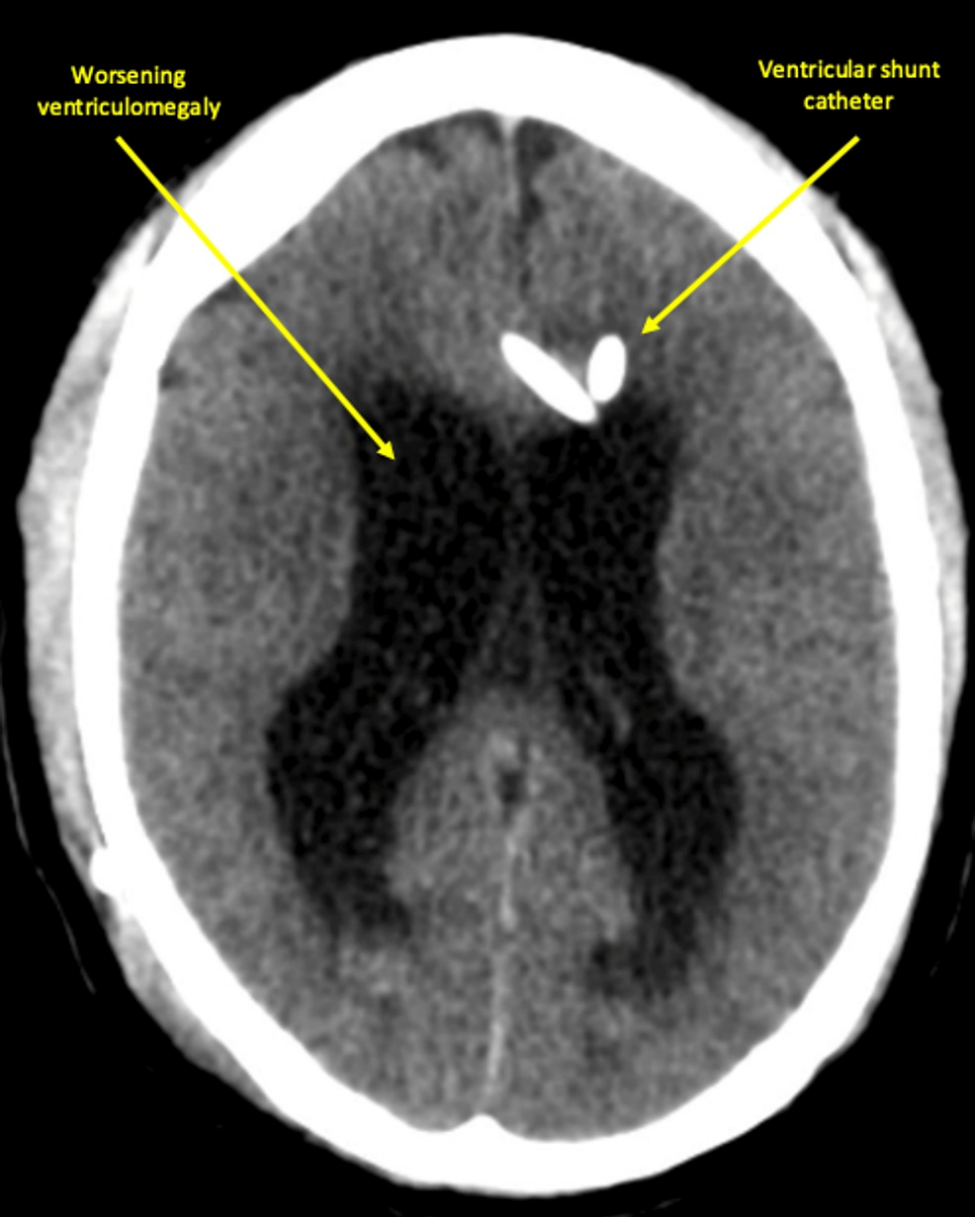
CT head without contrast on the first day of readmission displayed ventricular shunt catheter with new intraparenchymal hemorrhage at the right frontal lobe and extra-axial hemorrhage anterior to the left frontal lobe. In addition, worsening ventriculomegaly concerning for worsening hydrocephalus was also seen.

**Figure 4:**
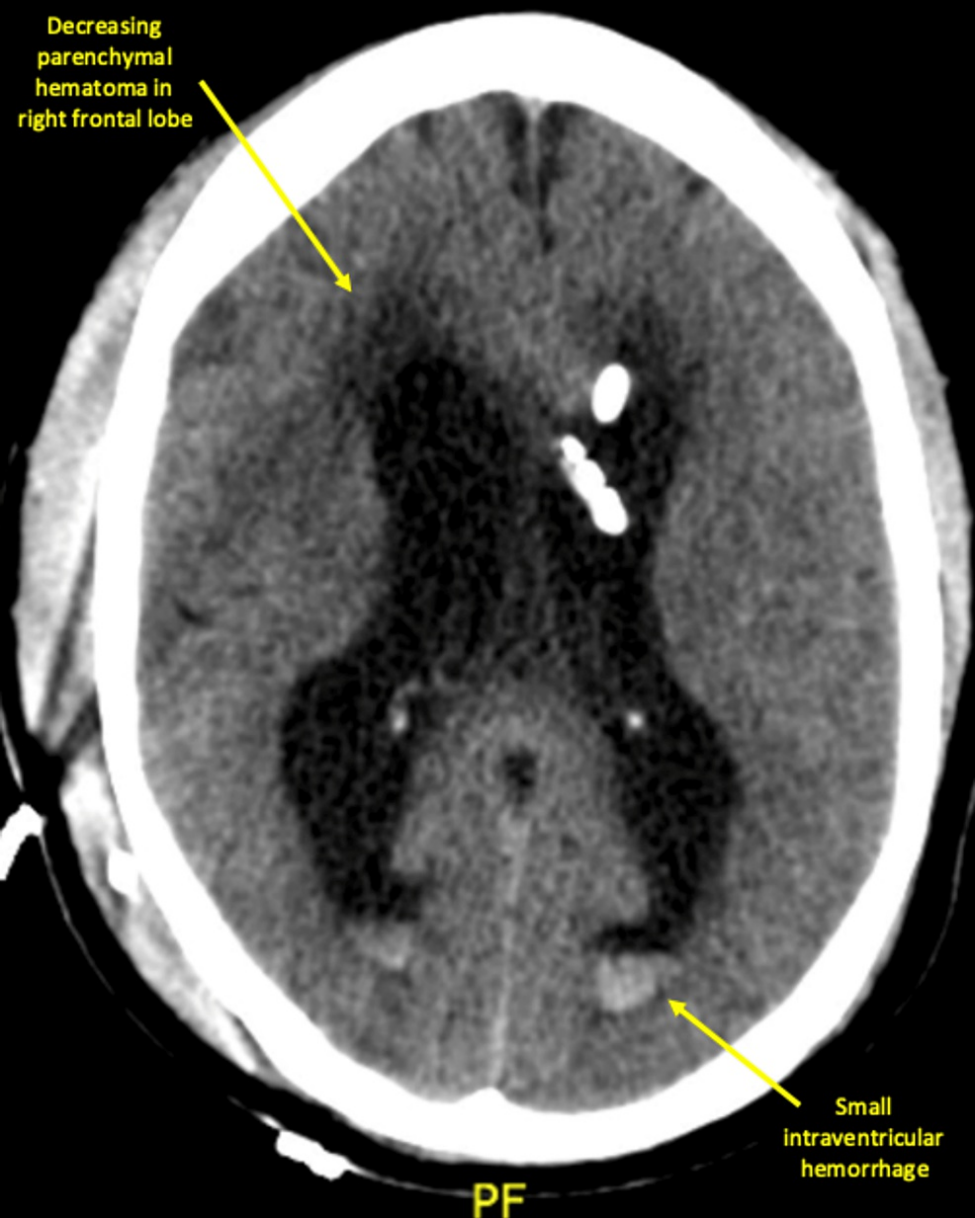
CT head without contrast on day 3 of the first readmission showed a decreasing parenchymal hematoma in the right frontal lobe adjacent to the shunt catheter but only a minimal decrease in size in the lateral and third ventricles. There was also a small amount of intraventricular hemorrhage and a small amount of pneumocephalus. “PF” represents the posterior fossa of the brain.

The patient returned to the hospital two days following discharge with altered mental status and apraxic gait. CT head showed a new 1.5 cm intraparenchymal hemorrhage at the right frontal lobe where the ventricular catheter was inserted and an extra-axial hemorrhage anterior to the left frontal lobe (Figure 3). There was also worsening ventriculomegaly concerning for hydrocephalus, and the patient was admitted to the ICU. Neurosurgery determined that the VP shunt was obstructed, and attempts to return patency were made by flushing the shunt through the proximal Ommaya reservoir. The patient also presented with a low sodium level of 126 mmol/L that was managed with 3% hypertonic saline and tolvaptan. The patient underwent a revision of the right frontal VP shunt with an external ventricular drainage catheter with a larger lumen and intake holes less prone to getting clogged. Postoperative CT head without contrast showed a decreasing parenchymal hematoma in the right frontal lobe adjacent to the shunt catheter (Figure 4). Over four postoperative days, the patient’s mentation improved, and he was stable from a neurosurgical standpoint. He was more communicative, with the greatest fluency in a spontaneous speech noted since his initial admission. The patient was alert and oriented to name, place, and month but not numerical date. Eight days after re-admission to the hospital, the patient was discharged with home oral medications; fluconazole 200 mg twice daily, two one gram tablets of sodium chloride three times daily, and oxycodone 325 mg-5mg every six hours as needed for pain. ​Nine days following discharge, the patient was seen in the neurosurgery outpatient clinic. He was found to have right-sided abdominal swelling with associated pain for the past three to four days. A kidney, ureter, and bladder (KUB) X-ray done outpatient revealed a distal shunt tube dislodged from the peritoneum into the subcutaneous tissue. The patient was then directly admitted to the hospital, where a surgical revision of the distal end of the shunt was completed, and the patient was discharged on hospital day three.

## Discussion

Coccidioidomycosis is caused by the pathogenic soil-dwelling dimorphic fungus, *Coccidioides immitis* (*C. Immitis*), or *Coccidioides posadasii* (*C. Posadasii*). *Coccidioides spp*. are specifically endemic to the Southwestern United States and parts of Mexico, Central, and South America. Of note, *C. posadasii* has a broad region of endemicity, from central and southern Arizona to western Texas and southern New Mexico [5]. Most Coccidioides infections occur in elderly persons, pregnant women, persons with immunodeficiency diseases, diabetics, transplant recipients, prisoners, and those of Filipino and African descent. Following inhalation, 60% of infected persons remain asymptomatic, and the majority of the remaining cases develop mild-to-severe symptomatic pulmonary infections [1]. However, only 1% of infected persons will develop disseminated disease involving the skin, joints, bones, CNS, or other organs [6]. The major risk factors for developing severe disseminated coccidioidomycosis include HIV/AIDS, immunosuppressive medications (i.e., prednisone, tumor necrosis factor (TNF) alpha inhibitors, chemotherapy, and organ transplantation medications), diabetes mellitus, pregnancy, and cardiopulmonary disease [6]. Disseminated disease can present with skin lesions, vertebral osteomyelitis, joint involvement, and CNS involvement. If left untreated, disseminated *Coccidioides* with CNS involvement is always fatal. Eosinophilic predominance is found in the CSF, and basilar meninges are often affected, leading to hydrocephalus as a common complication [1].

The prevalence of disseminated infections in immunocompromised individuals is well known. However, the association between disseminated infections of *Coccidioides* in immunocompetent individuals is less explored. A case report published in 2021 demonstrated an immunocompetent individual who succumbed to the disseminating effects of coccidioidomycosis. However, that patient presented with a bronchitis-like picture and progressively declined and developed septic shock with multi-organ system failure, requiring extracorporeal membrane oxygenation (ECMO) and an emergent bedside splenectomy [7]. That patient did not exhibit any CNS symptoms as were presented in our case. In addition, myelitis due to coccidioidomycosis was presented in a case of a 50-year-old immunocompetent patient with altered mental status, dizziness, headache, nausea, and quadriplegia. Following the investigation, the patient was diagnosed with acute coccidioidomycosis leading to meningitis and myelitis [8]. A case of rapidly progressing quadriparesis in the setting of disseminated coccidioidomycosis with cervical intramedullary involvement in an immunocompetent 55-year-old male has also been described. This patient presents similarly to ours as the absence of known coccidioidomycosis with an atypical presentation in an immunocompetent patient made the diagnosis elusive and challenging to discover in a timely manner [9].

Management of coccidioidal meningitis is based on antifungal treatment [10]. Oral fluconazole is typically the antifungal of choice, with itraconazole as an alternative. Our patient was originally on broad-spectrum antibiotic coverage with vancomycin, ampicillin, and ceftriaxone. However, following the diagnosis of coccidioidal meningitis, our patient was immediately started on IV fluconazole. If a patient responds well to the azole therapy, the treatment should be continued for life. In addition to medication management, if the patient has elevated intracranial pressure, repeat lumbar punctures may be warranted, and a shunt is typically necessary for patients who develop hydrocephalus. Due to hydrocephalus, our patient required a VP shunt. According to neurosurgery literature, there is a high likelihood that a VP shunt used for coccidioidomycosis will become clogged with debris and protein [11]. These difficulties can be manifested by a recurrence of headache, nausea, vomiting, gait disturbance, or mental status changes [12]. Following discharge, our patient returned to the hospital on two separate occasions with shunt complications, once with obstruction and once with dislodgement of the distal shunt. The VP shunt obstruction resulted in the patient’s altered mental status and apraxic gait, while the dislodgement was only associated with abdominal pain. Following these two separate admissions for shunt corrections, the patient was advised to follow up with neurosurgery and continue long-term medical management with oral fluconazole and oral sodium chloride.

## Conclusions

Coccidioidal meningitis can be confirmed via a positive serum *Coccidioides* antigen test and CSF analysis. It is treated with fluconazole, and it is important to note that for *Coccidioides* infection with CNS involvement, azole therapy must be continued for life. Additionally, hydrocephalus is a known complication of coccidioidal meningitis, and patients may require mechanical shunting with a VP shunt. This case demonstrates that although rare, coccidioidal meningitis should remain in the differential of a patient with altered mental status, evidence of meningitis even if the patient is immunocompetent. This case also highlights the importance of taking a thorough social history in patients with symptoms of disease outside of the expected geographical region for that particular microbiological etiology.
